# Poly(methyl methacrylate) in Orthopedics: Strategies, Challenges, and Prospects in Bone Tissue Engineering

**DOI:** 10.3390/polym16030367

**Published:** 2024-01-29

**Authors:** Susaritha Ramanathan, Yu-Chien Lin, Senthilkumar Thirumurugan, Chih-Chien Hu, Yeh-Fang Duann, Ren-Jei Chung

**Affiliations:** 1 Department of Chemical Engineering and Biotechnology, National Taipei University of Technology (Taipei Tech), Taipei 10608, Taiwan; susaritha21@gmail.com (S.R.); yuchienlin91@gmail.com (Y.-C.L.); senthilkumar2595@gmail.com (S.T.); f10421@mail.ntut.edu.tw (Y.-F.D.); 2 School of Materials Science and Engineering, Nanyang Technological University, Singapore 639798, Singapore; 3 Bone and Joint Research Center, Chang Gung Memorial Hospital, Linko, Taoyuan City 33305, Taiwan; 4 Department of Orthopaedic Surgery, Chang Gung Memorial Hospital, Linko, Taoyuan City 33305, Taiwan; 5 College of Medicine, Chang Gung University, Taoyuan City 33302, Taiwan; 6 High-Value Biomaterials Research and Commercialization Center, National Taipei University of Technology (Taipei Tech), Taipei 10608, Taiwan

**Keywords:** poly(methyl methacrylate), bone tissue engineering, bone cement, osseointegration, bioactivity

## Abstract

Poly(methyl methacrylate) (PMMA) is widely used in orthopedic applications, including bone cement in total joint replacement surgery, bone fillers, and bone substitutes due to its affordability, biocompatibility, and processability. However, the bone regeneration efficiency of PMMA is limited because of its lack of bioactivity, poor osseointegration, and non-degradability. The use of bone cement also has disadvantages such as methyl methacrylate (MMA) release and high exothermic temperature during the polymerization of PMMA, which can cause thermal necrosis. To address these problems, various strategies have been adopted, such as surface modification techniques and the incorporation of various bioactive agents and biopolymers into PMMA. In this review, the physicochemical properties and synthesis methods of PMMA are discussed, with a special focus on the utilization of various PMMA composites in bone tissue engineering. Additionally, the challenges involved in incorporating PMMA into regenerative medicine are discussed with suitable research findings with the intention of providing insightful advice to support its successful clinical applications.

## 1. Introduction

Bone is a highly vascularized, complex connective tissue with unique mechanical properties [1,2,3]. Bone tissue is dynamic and is constantly replaced by new bone through a continuous remodeling process. In addition, bone tissue performs numerous essential functions, such as providing structural support to the body, protecting internal organs, helping in movement, and bearing loads [4,5,6]. Structurally, bone comprises 50–70% inorganic phase and 20–40% organic phase, and the remaining is composed of water, lipids, and non-collagenous protein. The inorganic or mineral phase provides stiffness to the bone structure and consists of crystalline calcium phosphate in the form of hydroxyapatite (HAp). The organic phase comprises type I collagen, a fibrillar protein that provides mechanical support and acts as a framework for the mineralization process [7,8,9]. This meticulous organization of organic and inorganic phases imparts excellent mechanical properties and flexibility to bones.

Globally, the prevalence of bone disorders has increased recently, which can lead to socioeconomic crises and healthcare burdens [10,11]. Generally, bone-related diseases, such as fractures, bone defects, bone tumors, osteoarthritis, and osteoporosis, can occur as a result of trauma, injury, or age-related factors. These conditions can cause morbidity or discomfort in patients, severely affecting their quality of life [12,13]. Due to the self-healing capability of bone, minor bone defects can naturally repair themselves. However, if the extent of the defect is more significant, clinical intervention is required to treat the condition [14,15]. Thus, bone tissue reconstruction remains an essential but challenging task for both patients and surgeons.

Treatments such as metallic implants, autografts, and allografts are currently available to restore functionality and properly heal bone defects. These treatments are considered as “gold standard”; however, these treatments have their limitations. The use of metallic implants can lead to additional damage if they must be removed. The utilization of autografts is constrained by their limited availability, donor site morbidity, and surgical site infection, which can cause discomfort to patients. Similarly, the use of allografts is associated with a risk of disease transmission. Hence, it is necessary to develop a reliable treatment to address the unmet medical needs related to bone tissue defects and the limitations of current treatment modalities that can regenerate bone tissue without causing any discomfort to the patient [16,17,18]. Currently, several tissue engineering approaches are available for bone tissue regeneration. Tissue engineering is a rapidly evolving field that involves the use of cells, biomaterials, and bioactive agents to create biological substitutes or scaffolds that can help restore and promote the functions of specific tissues or organs [19,20].

In tissue engineering, the main requirement for a biomaterial is biocompatibility that does not produce any adverse reactions in the body [21]. Over the past few decades, a broad spectrum of materials intended for application in bone tissue engineering, including metals, ceramics, and polymers, have been studied. Polymers are frequently used in tissue engineering for various reasons such as their ability to tailor their mechanical properties and make them resemble the natural structure and properties of bone. Another important aspect is that customized scaffolds can be created for patients according to their needs because polymers provide flexibility in their processing methods. In most polymers, we can also modify their degradation rate according to the type of tissue, which is another important consideration [21,22,23,24].

Poly(methyl methacrylate) (PMMA) is a synthetic polymer that has attracted considerable attention in bone tissue engineering. PMMA is frequently used as a bone substitute and filler, and has a long history of successful use in orthopedic and dental applications, such as bone cement for joint replacement surgery. The major advantages of PMMA in bone tissue engineering include its biocompatibility, ease of handling, processability, and low cost. However, PMMA is bioinert and does not chemically bond or integrate with bone tissue at the implant site. Consequently, the host bone and bone cement exhibit weaker interactions. This type of inadequate bonding results in micromotion during regular activities. This could then enable tiny wear debris to form, which could induce osteolysis, followed by the aseptic loosening or potential displacement of the bone cement implant [25]. However, researchers have been exploring ways to improve the bioactivity and affinity of PMMA for bone tissue to enhance the long-term effectiveness of orthopedic operations. To enhance the biological activity of PMMA, it is often combined with various compounds such as HAp, carbon nanotubes, and natural polymers, including chitosan and collagen. Ni et al. developed bioactive strontium-containing HAp PMMA bone cement, which showed better bioactivity and osseointegration than pure PMMA bone cement after nine months of implantation in a goat revision hip replacement model [25,26,27,28,29,30,31].

**Scheme 1:**
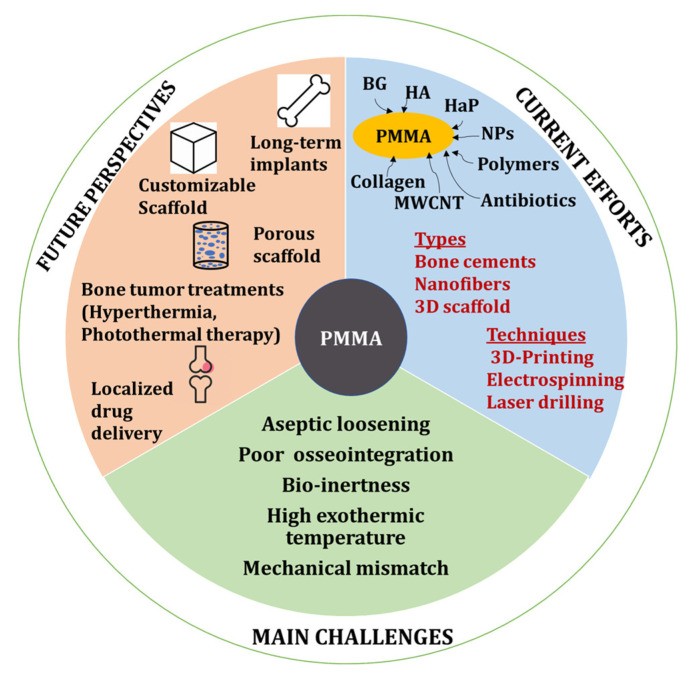
Current efforts, challenges, and future perspectives of PMMA in bone tissue engineering.

This review provides a comprehensive description of the use of PMMA in bone-tissue engineering. This review primarily focuses on the application of various PMMA composites including bone cement, scaffolds, and nanofibers in bone tissue engineering and the obstacles associated with integrating PMMA into regenerative medicine with relevant research findings, aiming to offer valuable insights and guidance to promote its effective clinical implementation (Scheme 1). Additionally, we discuss the physicochemical characteristics of PMMA and various polymerization techniques used to synthesize PMMA.

## 2. PMMA: A Comprehensive Exploration from Molecular Structure to Biomedical Applications

### 2.1. Physicochemical Properties

**Figure 1:**
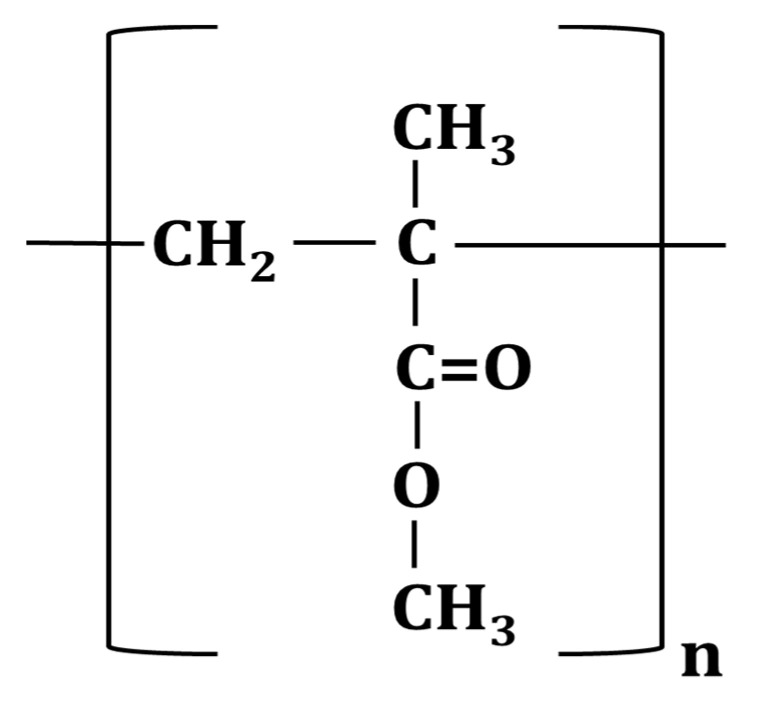
Structure of PMMA.

PMMA is a synthetic amorphous polymer derived from the monomer methyl methacrylate (MMA). Figure 1 shows the chemical structure of PMMA. Considering its hydrocarbon structure, it can be designated as poly [1-(methoxycarbonyl)-1-methyl ethylene], highlighting its composition in terms of hydrocarbon units. Alternatively, from an ester perspective, it is recognized as poly (methyl 2-methyl-propenoate), emphasizing the ester linkage present in its molecular makeup. The presence of a methyl group (CH_3_) within the polymer structure prevents tight packing in a crystalline manner and restricts free rotation around the C-C bonds [32]. PMMA, a thermoplastic material belonging to the acrylate family, is a highly transparent, colorless material that can be processed efficiently and is commonly used as a glass substitute because of its advantageous characteristics. PMMA has a high impact strength and is a lightweight but strong material that shows resistance to shattering. It exhibits compressive strengths ranging from 85 to 110 MPa, indicating its ability to withstand significant pressures. Furthermore, PMMA has an excellent tensile strength ranging from 30 to 50 MPa, indicating its resistance to stretching and pulling forces. The Young’s modulus of PMMA ranges from 2.4 to 3.3 GPa. PMMA has a glass transition temperature ranging from 100 °C to 130 °C, and its density is 1.20 g/cm^3^ at room temperature. The melting point at 130 °C allows PMMA to be easily molded and shaped when heated [32,33,34,35,36,37]. Regarding its interaction with moisture, PMMA exhibits a water absorptivity of 0.3%. In humid conditions, it reaches an equilibrium moisture absorption level ranging from 0.3 to 0.33%. Furthermore, during the molding process, PMMA displays a linear shrinkage mold ranging from 0.003 to 0.0065 cm/cm. PMMA stands out as one of the polymers renowned for its exceptional resistance to sunlight exposure, displaying minimal variation when subjected to UV radiation. Moreover, it exhibits significant thermal stability, capable of enduring extreme temperatures ranging from 100 to (−70) °C. PMMA exhibits outstanding optical properties beyond its robust physical attributes, with a refractive index of 1.490. Interestingly, PMMA does not degrade easily in an aqueous environment; therefore, it can be used for long-term implants [32,33,34,35,36,37]. The physicochemical properties of PMMA are summarized in Table 1.

### 2.2. Synthesis

**Scheme 2:**
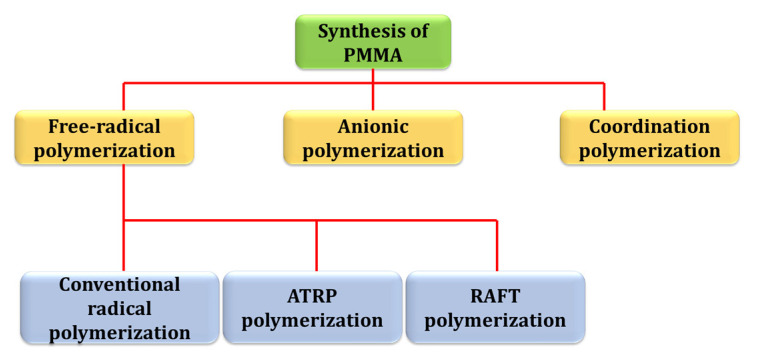
Synthesis of PMMA through various polymerization methods.

PMMA can be synthesized using several polymerization methods. As shown in Scheme 2, the monomer can undergo polymerization through commonly employed methods such as free-radical polymerization, reversible addition–fragmentation chain-transfer polymerization (RAFT), and atom transfer radical polymerization (ATRP). Anionic and coordination polymerizations can also be used to synthesize PMMA [38].

#### 2.2.1. Free-Radical Polymerization

MMA can undergo free-radical polymerization through various methods, including suspension, emulsion, solution, or bulk polymerization. During this process, MMA acts as the primary monomer, and an initiator is used. This initiator breaks down into free radicals when exposed to energy such as heat or light, depending on the chemical stability of the substances involved. These free radicals further interact with MMA, forming either an oligo radical or an initiation radical chain. During the termination step of free-radical polymerization, the active radical centers of the macroradicals are deactivated. This deactivation can occur through radical combinations, in which radicals join or transfer a hydrogen atom from one chain to another. This results in PMMA production [39]. Zhu et al. synthesized PMMA through the free-radical polymerization of MMA at 25 °C by employing 5 wt% of 2,2′-azobis(2-methylpropionitrile) (AIBN) as the initiator under ultraviolet light (UV) irradiation [40]. In another study, Kalra et al. reported the successful synthesis of PMMA from its monomer MMA through free-radical polymerization at ambient temperature by mixing a small amount of hydrogen peroxide and 2,4-pentanedione into a solution of water and a water-miscible solvent [41]. Christian et al. synthesized PMMA through the free-radical polymerization of MMA. Krytox 157FSL (DuPont, Wilmington, DE, USA), a commercially available acid-terminated perfluoropolyether, was used as a polymerization stabilizer in combination with supercritical carbon dioxide [42].

#### 2.2.2. Reversible Addition–Fragmentation Chain-Transfer Polymerization (RAFT)

RAFT is a type of radical polymerization that imparts living attributes to free-radical or conventional radical polymerizations. RAFT polymerization involves a reversible transfer mechanism between dormant species and a chain-transfer agent linked by a disulfide bond. This transfer process involves a cleavage addition reaction within a brief timeframe, resulting in the reduction of free radicals in the system. The advantage of RAFT polymerization is that it helps to synthesize a broad range of polymers with complex architecture, and their properties such as molecular weight and kinetics can also be controlled; the reaction can also be performed at low temperatures of 60 to 70 °C. This approach can be applied to various methods including bulk, solution, suspension, and emulsion polymerization [43]. Zhu et al. showed that MMA can be polymerized using the RAFT polymerization method employing 2-cyanoprop-2-yl 1-dithiophenanthrenate (CPDPA) as the RAFT agent [44].

#### 2.2.3. Atom Transfer Radical Polymerization (ATRP)

Using the controlled/living radical polymerization method known as ATRP, PMMA with a controlled molecular weight and narrow weight distribution can be produced. In ATRP, halogen-containing initiators such as alkyl or aryl halides are used along with a transition metal catalyst, typically copper or iron. To control the polymerization process precisely, the catalyst and initiator are combined to form a complex that reversibly activates and deactivates the expanding polymer chain. This technique can also be carried out in various forms, including bulk, solution, emulsion, and suspension polymerizations. It can be conducted at room temperature or higher depending on the specific catalyst and initiator used [45,46,47,48,49]. A previous study reported the polymerization of MMA using ATRP in the presence of 2-bromomethyl-4,5-diphenyloxazole as the initiator and CuBr/2,20-bipyridine (BPY) as the catalyst. The researchers used 1,4-Dioxane as a solvent and the reaction was carried out at an ambient temperature of 25 °C [46]. Another study reported the use of AIBN and tert-butyl peroxybenzoate (TBPB) as initiators for continuous activator regeneration (ICAR), and MMA was polymerized in bulk at a temperature of 70–120 °C [50].

#### 2.2.4. Anionic Polymerization

In the anionic polymerization of MMA, an anionic initiator, including an alkyl lithium compound such as n-butyllithium (n-BuLi) or sec-butyllithium, is used to initiate the reaction [51,52]. This reaction typically occurs in non-polar solvents such as anhydrous tetrahydrofuran (THF), which helps solubilize the reactants and controls the reaction rate [52,53,54,55,56]. During propagation, the carbonyl group of MMA is attacked by the anionic species produced by the initiator. As a result, a new carbon–carbon bond is formed, extending the polymer chain. The reaction does not stop until a termination step occurs or all the monomers are consumed. Numerous processes, such as coupling reactions, disproportionation, and interactions with impurities or termination agents, can result in termination. The choice of the termination mechanism can influence the molecular weight and polydispersity of the resulting polymer. The anionic polymerization of MMA typically produces high molecular weight polymers with narrow weight distribution and well-defined structures [52,53,54,55,56]. Ihara et al. reported the polymerization of MMA using the YCl_3_/lithium amide of secondary amine/nBuLi systems to produce PMMA at −78° C in THF [51]. Mita et al. reported the anionic polymerization of MMA in THF using a capillary flow method. They employed dianion of α-methylstyrene tetramer and sodium naphthalene as initiators [57].

#### 2.2.5. Coordination Polymerization

Coordination complexes are used as catalysts to initiate the polymerization reaction. The coordination polymerization of MMA can be carried out using various types of coordination catalysts, including titanocene, samarocene, cobalt (II) complexes, and group 4 cationic metallocene complexes. The coordination polymerization of MMA can be conducted in the presence of a co-catalyst such as triisobutylaluminum (TIBA) or MMAO, which can enhance the activity of the catalyst and control the properties of the resulting polymer [38,58]. Sun et al. reported the production of PMMA through the coordination polymerization of MMA, utilizing titanocene chloride and samarocene (Sm-Ti) as single-component catalysts, as well as triisobutylaluminum (TIBA), which acted as an activator [38].

The production of high-molecular-weight polymers is the primary benefit of conventional free-radical polymerization. However, obtaining polymers with a regulated structure and confined molecular weight distribution using this method is challenging. Apart from conventional free-radical polymerization, ATRP, and coordination polymerization, other techniques such as RAFT and anionic polymerization have been utilized to produce high-molecular-weight PMMA. However, there are limited reports on the synthesis of PMMA with a higher molecular weight using RAFT and anionic polymerization compared to free-radical polymerization. The mechanical characteristics of PMMA are significantly influenced by its molecular weight. It has been reported that the tensile strength, fracture surface energy, shear modulus, and Young’s modulus increase with increasing molecular weight up to 10^6^ Da. Compared with regular bone cement, bone typically has greater strength. Therefore, the use of PMMA with a higher molecular weight can result in the production of stronger PMMA bone cement. PMMA with viscosity-average molecular weights (Mv) in the range of 1.7 × 10^5^–7.5 × 10^5^ Da has proven successful in preparing PMMA bone cement, suggesting that high-molecular-weight PMMA holds promise as an excellent raw material for PMMA bone cement. Researchers have explored the correlation between the properties of PMMA denture bases and their molecular weight, revealing that denture base systems with weight-averaged molecular weights exceeding 10^5^ have optimal fracture strength properties. Therefore, PMMA with a higher molecular weight (Mn > 10^5^ Da) is a suitable candidate for dental materials [38].

### 2.3. Biomedical Applications of PMMA

**Figure 2:**
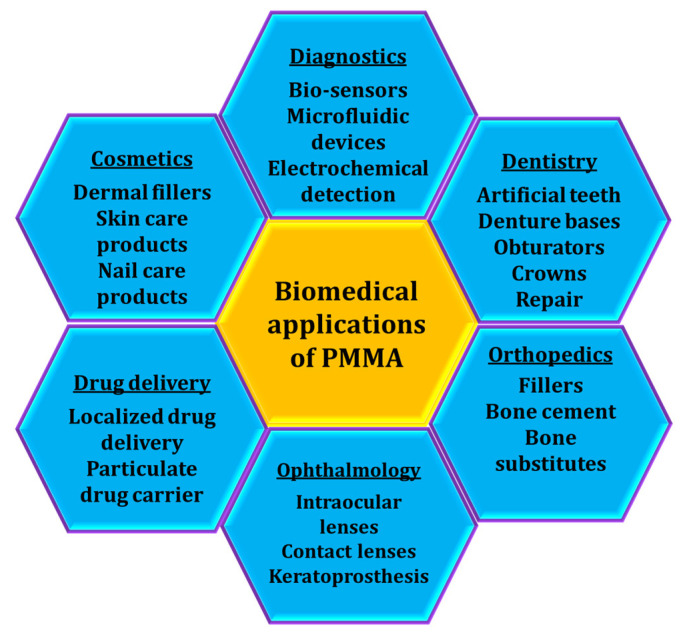
Biomedical applications of PMMA.

PMMA has attracted considerable interest in the field of biomedical engineering (Figure 2). Its widespread use can be attributed to its low cost, low density, simple processing, inert properties, cost-effectiveness, and ease of polymerization initiation. PMMA bone cement is frequently used to affix artificial joints to bones during orthopedic treatments, especially in joint replacement surgery. Additionally, it has been used as a bone cement to secure screws during bone fixation procedures to improve screw fixation and offer stability. PMMA bone cement has been used as a filler for bone cavities and defects, offering structural support and lowering the risk of fractures. PMMA is injected into cracked or weak vertebrae during vertebroplasty to stabilize them and reduce discomfort in patients with osteoporosis [59,60] In the past, PMMA was utilized to make intraocular lenses (IOLs), but it has now been replaced with more contemporary materials, such as silicone, because of its better properties [61,62]. PMMA is also used to fabricate contact lenses and keratoprostheses. PMMA microspheres are commonly used as dermal fillers in cosmetics. It is commonly used in skin care products such as lotions, face washes, and nail care products [63]. During drug delivery, PMMA can be used as a particulate drug carrier for various routes of administration [64]. It can also be used for localized drug delivery. It has also been used in microfluidic devices, biosensors, and electrochemical sensors [65]. It also has several purposes in dentistry, including the creation of dentures, fake teeth, denture bases, obturators, temporary crowns, and dental prostheses repair [32,66,67,68].

## 3. PMMA-Based Materials in Bone Tissue Engineering

### 3.1. PMMA Bone Cements

PMMA, also known as acrylic bone cement, is frequently used for bone substitution in orthopedic surgery. The utilization of PMMA bone cement is prevalent for the purpose of affixing prosthetic devices, including total hip or knee replacements, to the underlying bone structures [69,70,71,72]. Bone cement is prepared onsite in a surgical setting by combining two essential components. The first component is the liquid phase, which consists primarily of MMA. Additionally, the active ingredient N, N-dimethyl-p-toluidine (DMPT) is added to facilitate polymerization. This compound acts as a catalyst, effectively expediting the chemical reactions that convert the liquid monomer into a solid polymer. To maintain the stability of the liquid monomer and prevent undesired self-curing during storage, a stabilizer (hydroquinone) is carefully incorporated into the formulation. The second component, the powder phase, consists predominantly of PMMA particles, which form the fundamental framework of the cement. Benzoyl peroxide (BPO) is introduced to initiate polymerization upon mixing the powder and liquid components. This catalyst triggers a polymerization reaction, initiating a crosslinking process that transforms the liquid monomer into a solid polymer structure [73,74,75,76]. In addition to their primary constituents, bone-cement formulations commonly integrate radiopaque agents into the powdered phase. Zirconia (ZrO_2_) or barium sulfate (BaSO_4_) are commonly used for this purpose. These substances are essential for improving the cement visibility during medical imaging. Their inclusion enables the accurate monitoring and assessment of cement placement, distribution, and overall integrity following implantation [74,77].

The use of acrylic bone cement presents a multitude of advantages, including ease of preparation and application, rapid polymerization reaction, accelerated patient recuperation, and high compressive strength, and their final properties can be manipulated through the addition of other compounds to the cement. These advantages facilitate the efficient use of acrylic bone cements in surgical settings. Moreover, their ability to undergo swift polymerization allows immediate solidification, ensuring the prompt fixation of prosthetic devices. This in turn contributes to faster patient recovery and an expected return to normal activities. The comprehensive advantages offered by acrylic bone cement make it a valuable choice in orthopedic practice [78,79].

Cement exhibits excellent performance in fulfilling these functions, owing to its wide range of properties. However, it is widely acknowledged that bone cement has several drawbacks. The first drawback is that the high exothermic temperature of the cement (67 °C to 124 °C), at the center of the cement mantle, is believed to contribute to several detrimental effects. These include the thermal necrosis of the surrounding tissue, compromised local blood circulation, and the likelihood of membrane formation at the cement–bone interface. These effects are associated with the elevated temperature generated by cement during the polymerization process [80,81,82,83]. Various strategies have been employed to decrease the maximum exothermic temperature (Tmax) of PMMA bone cement, such as reducing the ambient temperature in the operating theater, cooling the cement powder or liquid before preparation [84,85], and lowering the molecular weight (M_w_) of the cement powder [86,87]. However, these approaches have shown only modest improvements. The incorporation of fillers into cement has been explored as another method to address this issue [88,89]. Yang et al. developed a novel PMMA composite for the treatment of percutaneous vertebroplasty (VP) and balloon kyphoplasty (BKP) by mixing PMMA bone cement with microcapsules containing a phase-change material (PCMc) (paraffin), which is highly effective in absorbing the heat produced during the polymerization process and may reduce thermal necrosis. Compared to the PMMA bone cement, the cement with 20% PCMc exhibited a notably reduced maximum exothermic temperature, prolonged setting time, and substantial reduction in both compressive strength and modulus. In vitro cell studies showed that the cement had good biocompatibility with L929 cells. To analyze the extent of thermal necrosis, cement was injected into fresh bovine lumbar vertebrae obtained from three 6-year-old healthy oxen. After 24 h after injection, PMMA with 20% PCMc showed a significantly smaller thermal necrosis zone than the pure PMMA cement [90].

Most MMA monomers in the PMMA bone cement undergo polymerization and become part of the solidified PMMA cement. However, a small amount of residual monomer may remain, and some may leak into the surrounding tissues or the surgical site, which is the second drawback. MMA release can occur during the initial setting phase of the cement and the subsequent curing process. The release of MMA from the PMMA bone cement is a concern because of its potential toxic effects on the surrounding tissues. MMA is known to have irritant properties and can cause adverse reactions such as tissue inflammation, allergic responses, and cytotoxic effects. The degree of toxicity depends on the concentration of released MMA, duration of exposure, and individual sensitivity [79].

Third, owing to its lack of bioactivity and limited osseointegration, PMMA bone cement can lead to certain issues. For example, the cement may separate from the surrounding bone over time, causing aseptic loosening or implant failure. Additionally, the lack of osseointegration can limit the ability of new bone to grow into the cement, potentially hindering the long-term stability of the implant. To address these limitations, researchers are actively exploring alternative materials and strategies for enhancing the bioactivity and osseointegration of bone cement. These include the incorporation of bioactive substances, such as HA or bioactive glass, nanomaterials, and other polymers, into the cement matrix, or the development of composite materials that combine PMMA with other bioactive components. These approaches aim to improve the biological response and integrate the cement with the surrounding bone, ultimately enhancing the long-term success of orthopedic implants [80,91].

**Figure 3:**
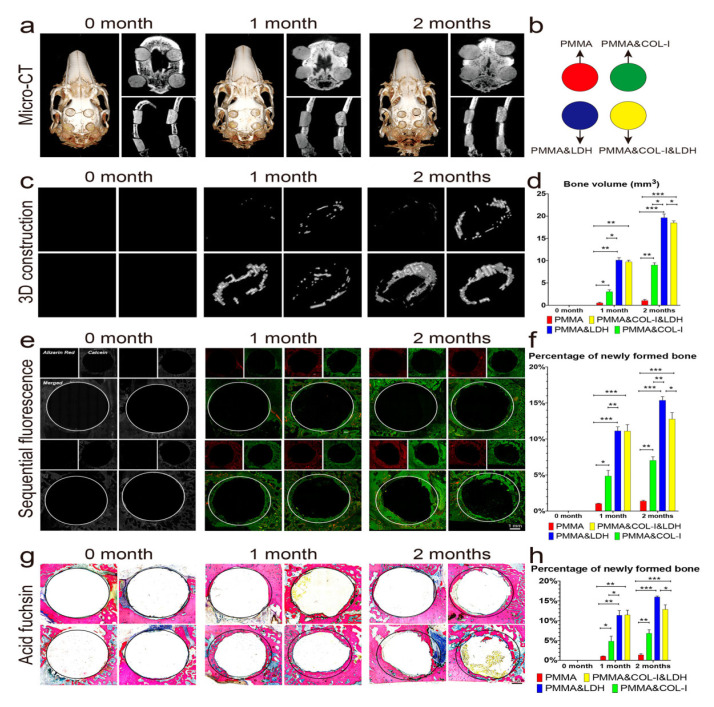
(**a**) Micro-computer tomography (µCT) images of cross-sectional views of 3D construction, sagittal, and coronal cross-section at 0, 1, and 2 months postoperatively. (**b**) PMMA and composites that were implanted in vivo. (**c**,**d**) Three-dimensional construction and quantitative analysis of newly formed bone tissues using micro-CT after 0, 1, and 2 months. (**e**,**f**) Representative views and quantitative analysis of new bone formation using sequential labeling analysis. (**g**,**h**) Acid fuchsin staining and quantitative analysis of new bone formation. Scale bar: 1 mm (e.g.,).* *p* < 0.05, ** *p* < 0.01, *** *p* < 0.001 (N = 3, one-way ANOVA followed by theStudent−Newman−Keuls post hoc test) [92].

Wang et al. developed a MgAl-layered double hydroxide (LDH) microsheet-modified PMMA (PMMA&LDH) bone cement to improve its osseointegration ability of the bone cement. The enhanced thermal insulation characteristics of the LDHs may impede thermal diffusion during the polymerization reaction of MMA, thereby safeguarding the surrounding osteoblast-related cells. In addition, magnesium ions released from LDHs can stimulate osteogenesis. Furthermore, the considerable size of the LDH microsheets can generate pores on the PMMA surface, promoting favorable osteointegration between the bone cement and bone. When compared to PMMA and PMMA&COL-I (mineralized collagen-I), the maximum polymerization reaction temperature of PMMA&LDH dropped by 7.0 and 11.8 °C, respectively. Compared to PMMA, the mechanical performance of PMMA&LDH marginally declined, which is advantageous for reducing stress-shielding osteolysis and, in turn, indirectly promoting osseointegration. In vitro cell studies suggested that the addition of LDH and COL-I enhanced the biocompatibility of PMMA bone cement in human bone marrow stem cells (hBMSCs). The LDH-modified PMMA bone cement had good osteogenic ability compared to the other groups. Additionally, transcriptome sequencing identified four key osteogenic pathways, p38 MAPK, ERK/MAPK, FGF, and TGF, in hBMSCs activated by PMMA&LDH. The results of in vivo studies shown in Figure 3 confirmed that the PMMA&LDH group promoted osseointegration and more new bone formation, and when compared to the PMMA&COL-I and PMMA groups, it increased bone growth in the New Zealand rabbit model by 2.17 and 18.34-fold, respectively [92].

**Figure 4:**
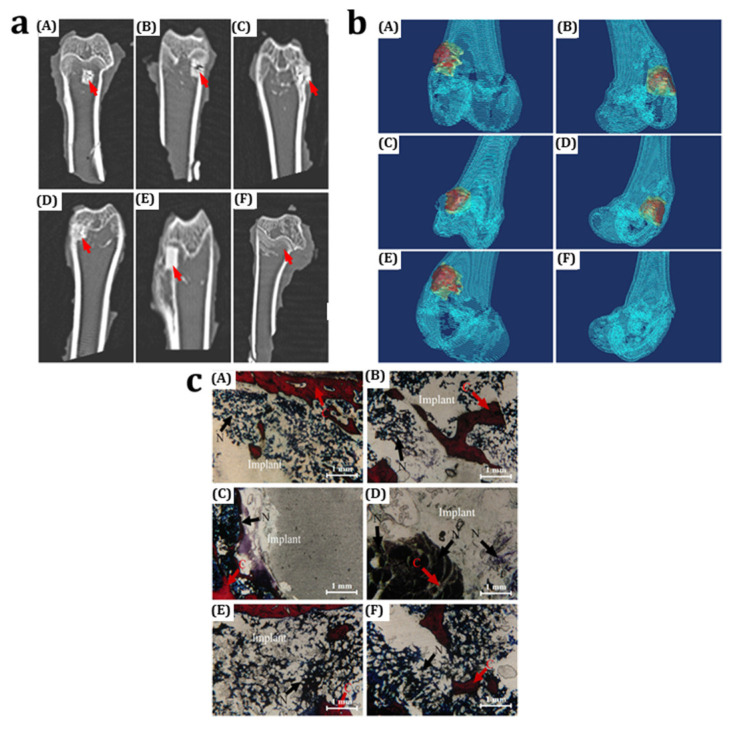
(**a**) CT images. The red arrow indicates implant position. (**b**) Three-dimensional reconstruction of CT images. (**c**) Van Giesen-stained images of PMMA-MWCNT bone cement specimens 12 weeks post implantation. Collagen fibers (C) are stained red. Nuclei (N) of osteoblasts are stained brown-black. In all the images (A)–(F) indicates the different PMMA-MWCNT bone cement groups (A) PMMA + 0.10 wt% MWCNT (B) PMMA + 0.25 wt% MWCNT (C) PMMA + 0.50 wt% MWCNT (D) PMMA + 1.0 wt% MWCNT (E) PMMA (Control) (F) Empty Defect [93].

Similarly, Wang et al. incorporated carboxyl-functionalized multi-walled carbon nanotubes (MWCNTs) into PMMA bone cement to improve its cytocompatibility and osseointegration. In vitro studies revealed that the incorporation of MWCNTs into PMMA led to increased DNA content as well as the upregulation of genes related to bone formation, such as osteonectin, osteopontin, and osteocalcin. The alkaline phosphatase/deoxyribonucleic acid (ALP/DNA) and protein/DNA ratios, which are indicators of osteogenic activity, were also higher in the presence of MWCNT-incorporated PMMA than in pure PMMA bone cement. Additionally, cell studies have suggested that the incorporation of MWCNTs into PMMA bone cement enhances the adhesion and proliferation of rat bone marrow stem cells (rBMSCs). The results of the in vivo study demonstrated that MWCNT powders increased bone mineral density and the number of osteoblasts in the cement implant material in a New Zealand rabbit bone defect model. However, there was a decrease in the number of collagen fibers, indicating a potential impact on the composition of the newly formed bone. Twelve weeks postoperatively, the bone cement resulted in new bone formation within the cement, as shown in Figure 4. Furthermore, the integration between bone cement and bone tissue was significantly enhanced as the MWCNT loading level increased [93].

**Figure 5:**
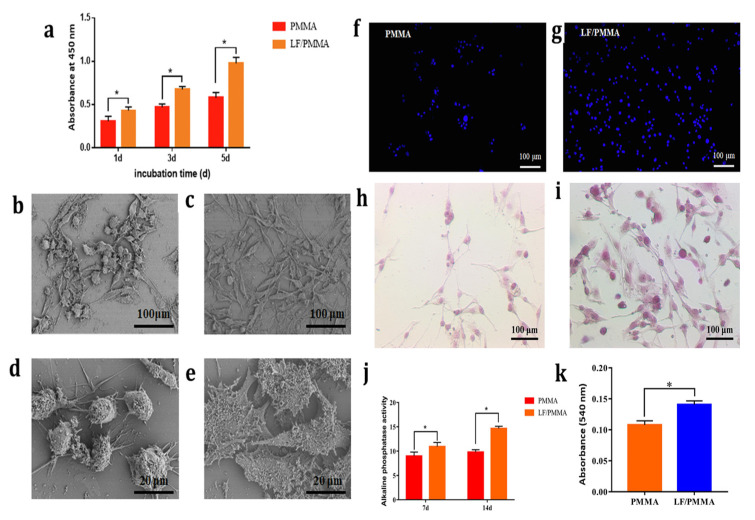
(**a**) Cell proliferation assay performed by culturing MC3T3-E1 cells on PMMA bone cement and LF-modified PMMA bone cement for 1, 3, and 5 d. Scanning electron microscopy (SEM) images of the MC3T3-E1 cells cultured on (**b**) PMMA bone cement and (**c**) LF-modified PMMA bone cement after 24 h incubation. SEM images of ECM secretion by MC3T3-E1 cells cultured on (**d**) PMMA bone cement and (**e**) LF-modified PMMA bone cement for 7 d. 4′,6-diamidino-2-phenylindole (DAPI) staining of MC3T3-E1 cell nucleus cultured on (**f**) PMMA bone cement and (**g**) LF-modified PMMA bone cement after 24 h of incubation. Calcium deposition performed using MC3T3-E1 cells on (**h**) PMMA bone cement and (**i**) LF-modified PMMA bone cement. (**j**) ALP activity of MC3T3-E1 cells cultured on PMMA bone cement and LF-modified PMMA bone cement. (**k**) Quantitative analysis of calcium deposition for 14 d. Asterisk (*) indicates significant differences (*p* < 0.05) [94].

In another study, Xu et al. modified the surface of PMMA bone cement with lactoferrin (LF) using chemical modifications and UV irradiation. The results of the in vitro studies shown in Figure 5 revealed that the LF-modified PMMA bone cement improved the cell adhesion, extension, and proliferation of mouse preosteoblast (MC3T3-E1) cells. Furthermore, ALP activity and extracellular matrix (ECM) mineralization were significantly enhanced after the modification of the surface of PMMA bone cement with LF [94].

The treatment of bone defects requires the use of materials or bone cements that possess superior mechanical properties and favorable biological characteristics. To address this challenge, this study focused on the synthesis and characterization of a novel PMMA bone cement comprising monticellite (Mon) and carbon nanotubes (CNTs) for effective bone-defect treatment. The addition of CNTs to the PMMA-Mon cement resulted in an enhanced resistance to cracking, which was attributed to the formation of bridges that effectively prevented crack propagation under additional stress. The in vitro bioactivity results suggested that PMMA bone cement containing Mon and CNTs exhibited greater apatite deposition than unmodified PMMA bone cement when immersed in simulated body fluid (SBF). Specifically, the incorporation of Mon and CNTs into the PMMA matrix facilitated the improved attachment of osteoblast-like cells (MG63) compared to PMMA cement, which demonstrates the non-toxic nature of bone cement [95].

Similarly, Boschetto et al. incorporated various concentrations (4, 5, 7.5, and 10 wt%) of curcumin into PMMA bone cement to improve its bioactivity. This study revealed that curcumin can be effectively combined with PMMA to create a homogeneous composite material across a wide range of concentrations, including concentrations up to 10 wt%. Increasing the percentage of curcumin in the composite material had a positive effect on cellular adhesion and bone production without compromising the quality of the formed bone tissue. However, adding curcumin beyond a threshold of approximately 5% led to a sudden decline in the ultimate strength while increasing the elongation to failure. In contrast, samples containing approximately 5% curcumin demonstrated favorable in vitro performance in KUSA-A1 cells without compromising their mechanical properties. These findings suggest that curcumin, in addition to its well-known antimicrobial activity, can serve as a cost-effective additive to improve the bioactive properties of PMMA, making it beneficial for bone regeneration [96].

Wekwejt et al. developed an antibacterial nanosilver-loaded PMMA cement (BC-AgNp) as an alternative to traditional cement for medical applications. However, the BC-AgNp lacks biodegradability and bioactivity. Therefore, BC-AgNp was modified by doping it with two extensively used bioactive glasses (BGs), silicate and borate, of various particle sizes. The addition of bioglass to BC-AgNp did not affect the polymerization process, although it led to a slight decrease in the mechanical properties and wettability of BC-AgNp. These findings demonstrate that BC-AgNp with smaller particles of melted BGs has higher porosity, a moderate deterioration of mechanical properties, and better antibacterial properties against *Staphylococcus aureus*, owing to its increased solubility. However, the cellular response may be negatively affected by the discharge of unreacted MMA monomers, which is the only drawback [97].

In this section, we discussed PMMA bone cement. Although PMMA is commonly employed as a bone filler and bone substitute material in orthopedic surgery, various studies have reported drawbacks of PMMA bone cement, such as high exothermic temperature and limited osseointegration, owing to the bioinert nature of the material. Therefore, long-term issues such as loosening may arise because of the lack of interaction between the material and tissues. Therefore, previous studies have attempted not only to overcome the drawbacks associated with PMMA bone cement but also contribute to its enhanced performance in orthopedic applications by incorporating various materials, and their outcomes are compiled in Table 2.

### 3.2. PMMA Nanofibers

The overall porosity of a bone tissue scaffold is crucial for encouraging the integration of a considerable number of osteogenic cells into the bone scaffold. Various studies have reported that structures with high porosities, interconnected pores, and large surface areas produce favorable results for tissue ingrowth. Among the various types of scaffolds, nanofibrous scaffolds have drawn considerable attention in bone tissue engineering because of their extraordinary characteristics that mimic the physical properties of the ECM. This property renders them suitable for promoting bone tissue growth and regeneration [103]. Numerous methods have been developed for fiber fabrication, such as electrospinning, wet spinning, melt spraying, and polymer cold drawing [103,104]. Among these techniques, electrospinning is a well-established and widely adopted approach for large-scale fiber production. Using this technique, fibers with diameters ranging from several nanometers to micrometers can be prepared continuously. Electrospinning has emerged as an exceptional technique for fabricating nanofibers owing to its simplicity, feasibility, versatility, and cost-effectiveness. It has several advantages, including the efficient production of nanofiber membranes with small pore sizes, high porosity, and significant specific surface areas. These qualities make electrospinning an attractive choice for tissue-engineering applications [104].

**Figure 6:**
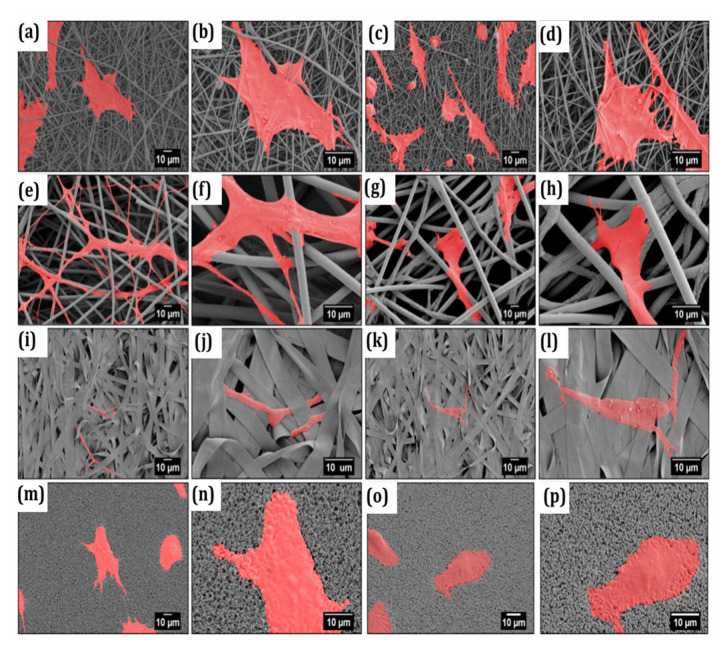
SEM images of MG63 cells (colored in red) on various PMMA scaffolds after 3 d of culture (**a**–**d**) nanofibers, (**e**–**h**) microfibers, (**i**–**l**) ribbons, and (**m**–**p**) film [105].

Ura et al. successfully prepared various types of electrospun scaffolds, such as nanofibers, microfibers, ribbons, and spin-coated films, using PMMA. In terms of surface properties, the PMMA films exhibited a smooth surface with a roughness (Ra) of <0.3 µm and displayed hydrophilic characteristics. In contrast, the fibers and ribbons demonstrated increased hydrophobicity, accompanied by a higher surface roughness and fiber diameter. The microfibers exhibited a contact angle of 140° due to their maximum roughness of 7 µm. In vitro cell studies have suggested that surface roughness is affected by fiber diameter. The increased roughness of the scaffold had a favorable effect on the attachment and interactions of MG63 cells, emphasizing the importance of surface features in encouraging positive cellular responses (Figure 6). The PMMA microfibers with an average fiber diameter exceeding 3.5 µm demonstrated a highly advantageous three-dimensional (3D) structure that promoted cell ingrowth within the scaffold. In contrast, for the other scaffold structures, the cells grew predominantly on the surface. This study revealed that the electrospinning of scaffolds with various geometries enabled the control of cellular development, allowing adjustments tailored to specific tissue requirements in the process of regeneration. These findings highlight the importance of the scaffold geometry in directing and influencing cellular behavior for successful tissue regeneration applications [105].

Taghiyar et al. fabricated keplerate polyoxometalate (Mo_132_)/metronidazole (MTN)/PMMA nanofibrous scaffolds using an electrospinning technique for guided bone regeneration (GBR) possessing antimicrobial activity and the ability to control drug delivery. The scaffolds obtained through electrospinning showed inherent characteristics such as outstanding tensile strength, high hydrophilicity (contact angle reduced from 126 ± 5.2° to 83.9 ± 3.2°, and water uptake increased from 14.18 ± 0.62% to 35.62 ± 0.24%), appropriate bioactivity, and cell adhesion after the addition of Mo_132_ and metronidazole. Furthermore, the biodegradation rate of the resulting scaffolds was enhanced by the inclusion of Mo_132_ and metronidazole, surpassing that of pure PMMA membranes. The proliferation rate of MG-63 cells improved after 7 d of culture for Mo_132_/MTN/PMMA compared to the pure PMMA scaffold. Owing to the regulated release of metronidazole over 14 d, a noticeable inhibitory zone was observed that prevented the growth of *Escherichia coli*. These findings indicate that Mo_132_ and MTN-loaded PMMA scaffolds have great potential for bone regeneration [106].

Xing et al. fabricated PMMA/HAp nanofibers using an electrospinning technique by incorporating two different concentrations of HAp nanoparticles (10% and 20%) into a PMMA solution. In vitro cell studies showed that the number of osteoblasts present on the PMMA/HAp nanofibrous scaffolds was noticeably higher than that on the pure PMMA scaffolds. In addition, the osteoblasts on the PMMA/HAp nanofibrous scaffolds exhibited improved cytoskeletal organization and higher ALP activity than those on the PMMA control. Moreover, the release of calcium ions from the PMMA/HAp nanofibrous scaffolds containing 20 wt% HAp (PMMA/HAp20) was notably higher than that from the scaffolds with 10 wt% HAp (PMMA/HAp10). These results strongly suggest that the inclusion of HAp in PMMA nanofibrous scaffolds enhances osteoblast differentiation [107].

Nanofibrous scaffolds offer several advantages, such as high porosity, large surface area, and a structure that mimics the natural ECM. These properties render them suitable for bone tissue engineering applications. PMMA is traditionally used in bone cement, rather than as a material for tissue engineering. Currently, there are a limited number of research articles on PMMA nanofibers for bone tissue engineering; however, future developments in these areas could contribute to a renewed interest in exploring PMMA nanofibers for bone tissue engineering. Previous studies on PMMA nanofibers for bone tissue engineering applications are summarized in Table 3.

### 3.3. Three-Dimensional PMMA Scaffolds

In tissue-engineering applications, 3D scaffolds play a crucial role in creating an optimal microenvironment for the integration of cells and growth factors, facilitating the regeneration of damaged tissues and organs. The main advantage of 3D scaffolds is that they mimic the authentic in vivo microenvironment, enabling cells to interact with and respond to mechanical signals derived from their neighboring surroundings. Three-dimensional scaffolds can be tailored to meet individual patient requirements, encompassing dimensions such as size, shape, and porosity, a level of customization that is not achievable with bone cement. These scaffolds can be designed to have physical and mechanical properties that mimic those of native tissue, providing better support for tissue regeneration; however, bone cement has limited mechanical properties [108,109,110,111].

Matbouei et al. developed a 3D scaffold using PMMA and investigated its structural and mechanical properties. The fabrication process involved the use of a laser to cut the PMMA sheets into a specific pattern; the sheets were then stacked and bonded together to form a porous interconnected structure. To improve the bioactivity of the scaffold, a thin layer of chitosan/bioglass was coated on its surface. In vitro studies showed that the chitosan/bioglass-coated scaffold had larger cell proliferation and increased bioactivity when compared to the pure PMMA scaffold [112].

Baura et al. developed HAp/PMMA-based porous scaffold by incorporating different concentrations of zinc oxide (ZnO) nanoparticles for bone tissue engineering applications. It was found that the inclusion of ZnO up to 5% (*w*/*w*) greatly improves the porosity, compressive strength, thermal stability, and swelling characteristics of the scaffold. The addition of 5% (*w*/*w*) ZnO to the composite improved the biodegradability and in vitro bioactivity of the scaffold in SBF. The results of the in vitro cytotoxicity test performed in MG63 cells showed that the composite scaffold had good biocompatibility and found to be a suitable candidate for bone tissue engineering [113].

Rahmani-Monfard et al. developed a novel approach for creating 3D scaffolds from PMMA, using a CO_2_ laser drilling technique by employing a computer-controlled laser drilling machine to obtain arrays of interconnected holes with predetermined patterns and geometries on bulk PMMA samples. The results showed that this method is superior to other approaches in that it enables the creation of scaffolds with a high degree of interconnectivity and controllability of the porosity, pore size, and mechanical characteristics. To boost their bioactivity, the fabricated samples underwent a surface coating process with a thin layer of chitosan/β-tricalcium phosphate (β-TCP) composite. Moreover, the chitosan/β-TCP composite coating enhanced the interaction between osteoblast-like cells and the polymeric scaffolds, leading to an accelerated rate of cell proliferation [28].

**Figure 7:**
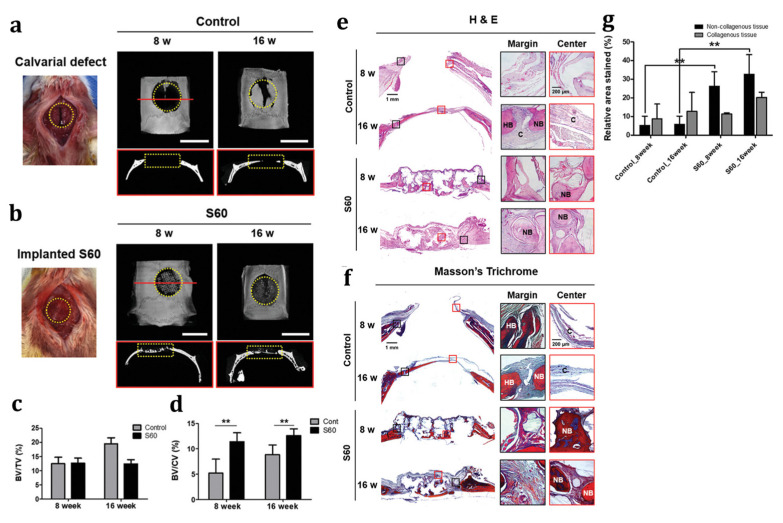
(**a**,**b**) Representative photograph of critical size calvarial defect (φ 8 mm highlighted with yellow dotted line) in SD rat model, and micro-CT images of 3D and coronal (2D slice along the red line of 3D image) at 8 and 16 weeks after the implantation of the scaffold. (**c**) Morphometric analysis of the volume of newly formed bone volume (BV/TV) relative to total skull defect volume. (**d**) Ratio of newly formed bone volume to central volume (BV/CV). Histological evaluation of calvarial bone regeneration at 8 and 16 weeks after implantation. (**e**) Hematoxylin and eosin (H&E) staining. (**f**) Masson’s trichrome (MT) staining of calvarial bone defects. (**g**) Quantitative analysis of mature bone tissue formation based on MT staining images. ** indicates *p* < 0.01 [114].

For the first time, 3D printing of an inorganic–organic hybrid scaffold was developed using the star polymer poly (methyl methacrylate-co-3-(trimethoxysilyl)propyl methacrylate) and silica, which exhibited promising mechanical properties. The 3D printing process involves the direct ink writing of the sol, resulting in bone substitutes with various inorganic–organic ratios of poly (methyl methacrylate-co-3-(trimethoxysilyl) propyl methacrylate)-star-SiO_2_ hybrid inks and pore channels sized between 100 and 200 µm. The 3D-printed scaffolds possess mechanical properties that closely mimic those of the trabecular bone. In vitro experiments demonstrated that MC3T3 preosteoblast cells adhered to the scaffolds, irrespective of their composition. Further studies using a rat calvarial defect model revealed the osteogenic and angiogenic properties of the hybrid scaffolds (Figure 7). In particular, scaffolds with a 40:60 inorganic–organic composition have demonstrated significant capabilities in promoting new vascularized bone formation within their pore channels. Additionally, these scaffolds induce macrophages to adopt the M2 phenotype that supports tissue repair and regeneration [114].

Although PMMA scaffolds possess several advantages, their lack of inherent bioactivity and challenges in promoting cell adhesion and tissue integration limit their use in tissue engineering applications. Researchers have often explored surface modifications, the incorporation of bioactive agents, or composite materials to address these limitations and enhance the overall performance of PMMA scaffolds in tissue engineering applications. Previous studies on PMMA scaffolds are listed in Table 4.

## 4. Future Perspectives

PMMA has been widely used in various medical applications, including orthopedics and dentistry. It is not a traditional biomaterial used in bone tissue engineering; however, it is primarily used as bone cement in this field. PMMA is biocompatible in nature but is not a biologically active material. The use of bone cement has several disadvantages, including a high exothermic temperature, MMA release, poor osseointegration, and the aseptic loosening of the implant. Researchers have explored various strategies to overcome these drawbacks and improve the overall performance of bone cement by incorporating bioactive compounds into PMMA. Nanofibers, typically produced using techniques such as electrospinning, offer structural resemblance to the extracellular matrix and provide a biomimetic environment for cell attachment and growth. In contrast, scaffolds serve as 3D frameworks that support cell adhesion, proliferation, and differentiation. Few studies have explored PMMA nanofibers and 3D scaffolds. However, a significant gap persists in conducting comprehensive in vivo experiments to confirm the feasibility of these scaffolds. Although significand research has been conducted on PMMA bone cement, and is still ongoing, it is necessary to explore other techniques such as porous scaffolds, nanofibers, and 3D scaffolds on PMMA to better utilize this material in the field of tissue engineering. Combining PMMA with biomaterials such as HAp, calcium phosphate, or biodegradable polymers can influence the mechanical properties of PMMA and improve its bone regeneration ability. Recently, the ability to design scaffolds and implants specifically for patients has become possible with the help of 3D printing technology. PMMA can be employed as a material in 3D printing techniques, enabling the creation of bone implants that are specifically tailored to user needs. Thus, PMMA can be used as a carrier for localized drug delivery. A few researchers have reported the use of PMMA for releasing compounds such as curcumin, gentamycin, and antibacterial silver nanoparticles. The use of PMMA implants that release growth factors, antibiotics, or other bioactive compounds should be investigated by researchers as options to promote bone healing in the surrounding tissue and prevent infection. Similarly, for the treatment of bone tumors, the incorporation of magnetic nanoparticles or photothermal agents into the PMMA scaffold and the application of an external magnetic field/irradiation with NIR light to generate localized heat selectively destroy tumor cells while minimizing damage to surrounding healthy tissue. This approach can be explored for its potential benefits in localized and targeted therapies. PMMA is an inherently non-degradable material, making it suitable for long-term implants. However, the degradation rate can be varied by combining PMMA with other compounds, according to the type of tissue. Surgical removal is unnecessary because these materials would progressively deteriorate and are replaced by newly produced bone tissue. As the field of tissue engineering continues to evolve, the further exploration of PMMA, particularly in conjunction with innovative techniques, holds promise for achieving breakthroughs in regenerative medicine.

## 5. Conclusions

PMMA is considered a promising material in the field of bone tissue engineering for various applications, including bone cements, fillers, and bone substitutes. Although the use of PMMA has various advantages such as biocompatibility, affordability, and flexibility, it also has disadvantages such as a lack of bioactivity. Various strategies have been used to enhance its biocompatibility and bioactivity. However, it is important to note that further research and development is needed to optimize its properties for specific applications and address its potential limitations. The biocompatibility and long-term performance of PMMA-based constructs must be thoroughly evaluated to ensure their safety and effectiveness in clinical settings.

## Figures and Tables

**Table 1:** Physicochemical properties of PMMA.

Properties	PMMA	Reference
Type	Synthetic	[32]
Color	Colorless	[32,36]
Compressive Strength	85–110 MPa	[32]
Tensile Strength	30–50 MPa	[32]
Glass Transition Temperature	100–130 °C	[26,32,37]
Density	1.20 g/cm^3^	[32,36,37]
Melting Point	130 °C	[32,36]
Water Absorptivity	0.3%	[32,36]
Moisture Absorption Level	0.3–0.33%	[32,36]
Linear Shrinkage	0.003–0.0065 cm/cm	[32]
Young’s modulus	2.4–3.3 GPa	[36]
Refractive Index	1.490	[32,36]

**Table 2:** List of PMMA composite bone cements for bone tissue engineering.

Composition		Additives	In Vitro	In Vivo	Reference
Liquid phase: MMA	Powder phase: PMMA	PCMc (paraffin)	Cell type: L929 cells Excellent biocompatibility	Animal model: 6-year-old oxen Significantly smaller thermal necrosis zone	[90]
Liquid phase: MMA	Powder phase: PMMA	COL-I MgAl-LDH	Cell type: hBMSC Good biocompatibility Excellent osteogenic ability	Animal model: New Zealand white rabbits Promoted osseointegration More new bone growth formation	[92]
Liquid phase: MMA DMPT Hydroquinone	Powder phase: PMMA BPO BaSO_4_	Carboxyl functionalized MWCNT	Cell type: rBMSC Promoted cell adhesion, proliferation, and osteogenic gene expression	Animal model: Male New Zealand white rabbit Promoted osteointegration Increased bone mineral density and collagen fiber content	[93]
Liquid phase: MMA	Powder phase: PMMA	LF Carbodiimide hydrochloride (EDC)	Cell type: rBMSC Promoted cell proliferation, adhesion, and extension Promoted mineralization, ECM secretion, and ALP activity	None	[94]
Liquid phase: MMA	Powder phase: PMMA	Monticellite Carbon nanotubes (CNT)	Cell type: MG63 Enhanced cell viability Promoted cell adhesion and extension	None	[95]
Liquid phase: MMA DMPT ethylene glycol dimethacrylate	Powder phase: PMMA Copolymer of methacrylate BPO	Curcumin	Cell type: KUSA-A1 cells Promoted cell adhesion Increased ECM secretion	None	[96]
Liquid phase: MMA Dimethyltryptamine (DMT)	Powder phase: PMMA BPO BaSO_4_	HAp β-tricalcium phosphate (β-TCP)	Cell type: rBMSC Promoted adhesion, differentiation, and proliferation	Animal model: New Zealand rabbit Promoted more new bone growth	[98]
Liquid phase: MMA DMPT Hydroquinone	Powder phase: PMMA BaSO_4_ BPO	Bioglass AgNP	Excellent antibacterial activity against *S. aureus*	None	[97]
Liquid Phase: MMA DMPT Hydroquinone	Powder phase: PMMA	Gelatin Gentamycin sulfate	Cell type: rBMSC Promoted cell attachment, proliferation, and differentiation Bacterial species: *S. aureus* and *E. coli*	None	[99]
Liquid phase: MMA	Powder phase: PMMA	Nano MgO particle	Cell type: MC3T3-E1 cells Excellent biocompatibility Formation of more calcium nodules High expression of osteogenic markers	Animal model: 6-week-old SD rats More new bone growth Bone mineral density was 50% higher than that of PMMA bone cement	[100]
Liquid phase: MMA γ-methacryloxypropyltrimethoxysilane (MPS) DMPT	Powder phase: PMMA BaSO_4_ BPO	Calcium acetate	None	Animal model: Male Japanese white rats After 4 weeks, osteoconduction was observed at the modified bone cement	[101]
Liquid phase: MMA	Powder phase: PMMA	Chitosan or hydroxypropyl trimethyl ammonium chloride chitosan, (HACC) powder Gentamycin	Cell type: hMSCs Good Biocompatibility High ALP activity Higher expression of osteogenic markers like Collagen-1, osteopontin, osteocalcin, and ALP	None	[102]

**Table 3:** List of PMMA composite nanofibers for bone tissue engineering.

Components	Technique	In Vitro	In Vivo	Reference
PMMA	Electrospinning and spin coating	Cell type: MG63 cell lines Promoted cell attachment	None	[105]
Mo_132_/MTN/PMMA	Electrospinning	Cell type: MG63 cell lines Promoted cell adhesion on the surface	None	[106]
PMMA/HAp	Electrospinning	Cell type: MC3T3-E1 Promoted cell adhesion and proliferation Increased ALP activity	None	[107]

**Table 4:** Various 3D-PMMA composite scaffolds for bone tissue engineering.

Material	Method of Fabrication	Cellular Assay	In Vivo Experiment	Reference
PMMA with chitosan/β-TCP coating	CO_2_ laser drilling technique	Cell type: SaOS-2 cells Significantly higher cell proliferation rate	None	[28]
PMMA with chitosan/bioglass composite coating	Layer cutting by CO_2_ laser	Cell type: SaOS-2 cells High cell proliferation rate	None	[112]
Star polymer poly (methyl methacrylate-co-3 (trimethoxysilyl)propyl methacrylate) and silica	3D-printing	Cell type: MC3T3-E1 Good cell attachment	Animal model: SD rats Promoted new bone growth formation after 16 weeks	[114]
PMMA-nHAp	Conjugated TIPS and wet-chemical route	Cell type: SaOS-2 cells High cell proliferation rate	None	[115]
Polyhydroxyethylmethacrylate/PMMA	Chemical synthesis	None	Animal model: New Zealand white rabbit Promoted more new bone growth after 8 weeks.	[116]
PMMA-Calcium Phosphate	Thermal-induced phase separation method (TIPS)	Cell type: MG63 cells Excellent cytocompatibility and hemocompatibility	None	[117]

## Data Availability

Not applicable.
